# Supplementary material for: Deciphering metabolic differentiation during Bacillus subtilis sporulation
Source: Nat Commun. 2025 Jan 2;16:129. doi: 10.1038/s41467-024-55586-z (PMC11695771; doi:10.1038/s41467-024-55586-z)
Supplement: Supplementary file 1 — Supplementary Information [file 41467_2024_55586_MOESM1_ESM.pdf]

Supplementary Information for

Deciphering metabolic differentiation during *Bacillus subtilis* sporulation

Juan D. Tibocha-Bonilla & Jelani Lyda *et al*.

*Corresponding authors. Email: (Karsten Zengler) kzengler@ucsd.edu; (Kit Pogliano) kpogliano@ucsd.edu.

This PDF file includes:

Supplementary Figures 1 to 5

Supplementary Tables 1 to 4

Supplementary Methods

Supplementary Figure 1. Breakdown of (a) reactions and (b) metabolites in *SporeME2*.

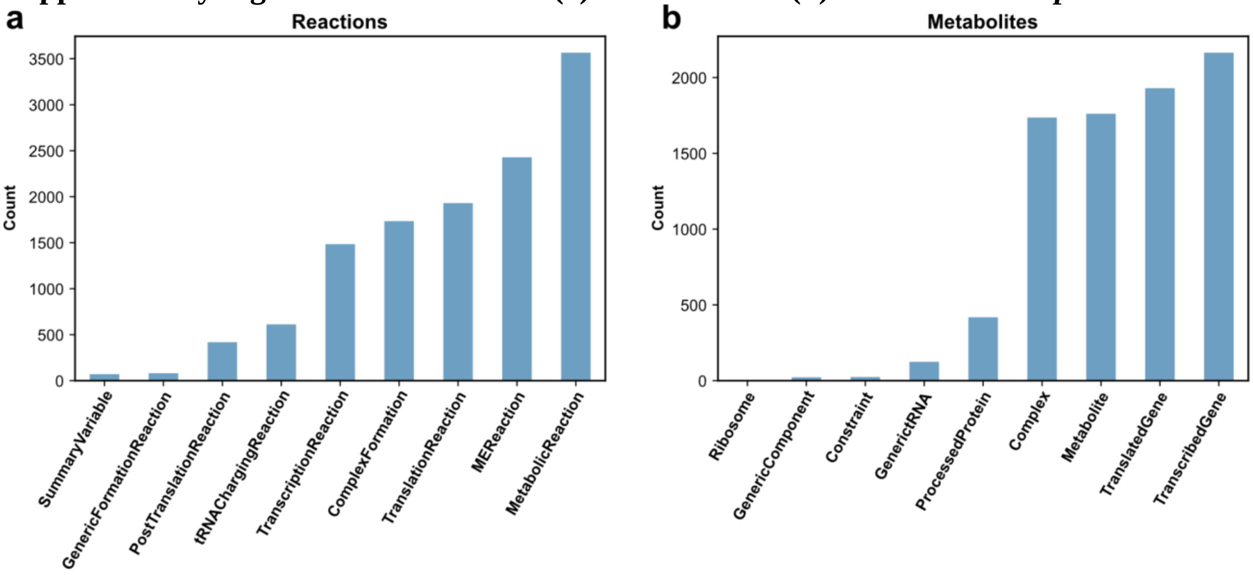

Supplementary Figure 2: Localization of enzymes during sporulation. Imaged 3 hours after sporulation initiation. Membrane in red, GFP-tagged enzyme in green. Scale bar = 1 ɥm.

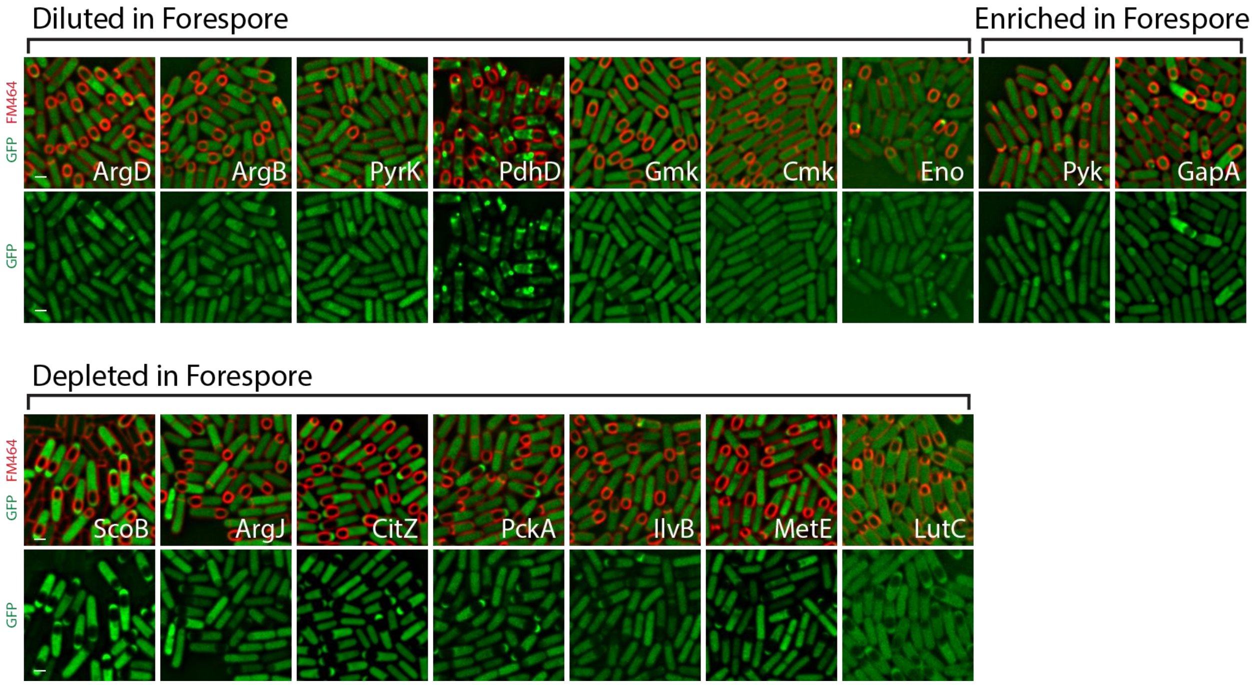

Supplementary Figure 3: Degradation of Cmk and PyrG alone show no sporulation phenotype. a, Membrane fusion assay of *cmk-ssrA* and *pyrG-ssrA* mutants at 3, 4 and 5 hours after sporulation initiation using fluorescent and phase microscopy. The tagged proteins were degraded using the STRP system in either the mother cell only [BJAL134, BJAL138] the forespore only [BJAL082, BJAL084], or in both compartments [BJAL136, BJAL140] and compared to the -*ssrA* tagged only strain [KP1602, KP1734]. The membrane is red and green. Phase bright endospores are indicated by double white arrows. No sporulation defect was observed. Scale bar = 1 ɥm. **b,** Spore titer of *cmk-ssrA* and *pyrG-ssrA* mutants 24 hours after sporulation initiation. *PyrG-ssrA* shows a slow germination phenotype, likely because PyrG is essential in *B subtilis* vegetative cells, so the germinating cells need to compensate for its loss (Supplementary Table 1). **c,** Membrane staining of *cmk-ssrA* + *pyrG-ssrA* double mutant at 3, 4, and 5 hours after sporulation initiation as shown in **Figure 2d**. Proteins were degraded using the STRP system in either the FS, MC, or both compartments. Membrane in red and green. Mid-engulfment cells are indicated by the single yellow arrow. FM4-64 only staining more easily reveals the sporulation delay cause by degradation of Cmk and PyrG in the MC. A lack of CDP and UDP in the both degradation strain causes lysis in the cells, as seen by the double yellow arrows. Scale bar = 1 ɥm.

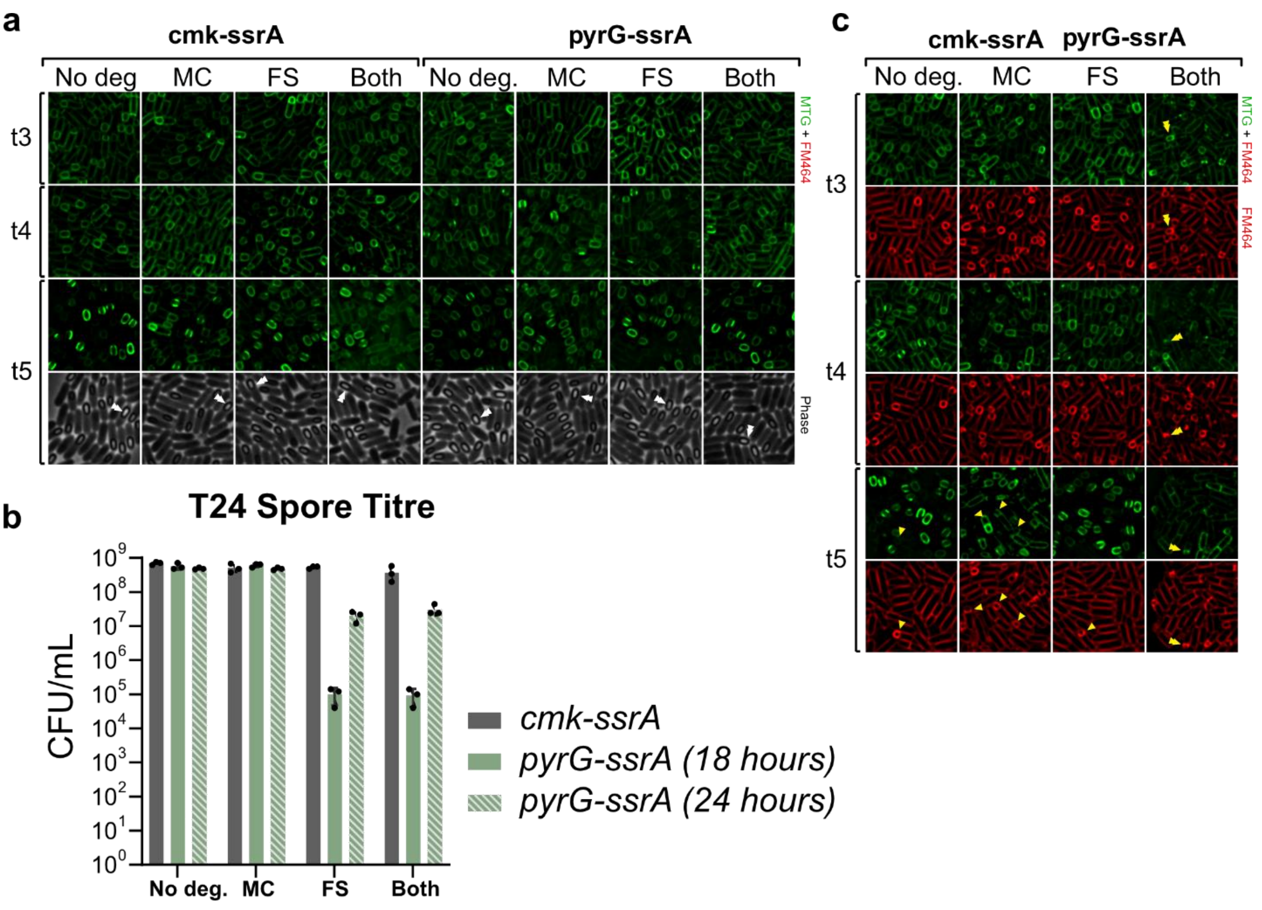

Supplementary Figure 4: ATP production in the forespore if ATP is allowed to be transported from the mother cell. ATP is fed to the forespore, which supplied directly 50% of its requirement for biosynthesis, 49.6% is supplied by ADP interconversion to ATP. This ADP is produced by ATP hydrolysis due to maintenance, which is also fueled by ATP transport. Therefore 99.6% of ATP supply in the forespore is supplied by ATP transport, if allowed.

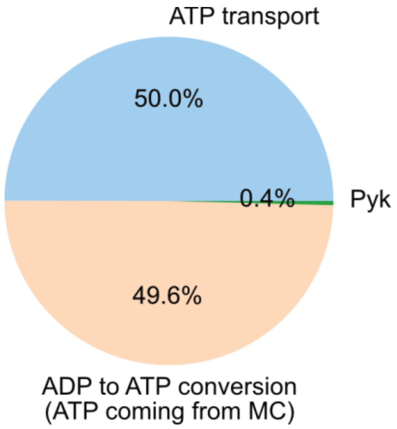

Supplementary Figure 5: Degradation of Pyk and GapA show no sporulation phenotype. a, Membrane fusion assay of *gapA-ssrA* and *pyk-ssrA* mutants at 3, 4 and 5 hours after sporulation initiation using fluorescent and phase microscopy. The tagged proteins were degraded using the STRP system in either the mother cell only [BJAL142, BER1784] the forespore only [BER0021, BER1785], or in both compartments [BJAL144, BER1786] and compared to the *-ssrA* tagged only strain [BER0002, BER1786]. The membrane in red and green. Phase bright endospores are indicated by double white arrows. No sporulation defect was observed. Other ATP generation pathways are potentially compensating for the loss of an individual protein. Scale bar = 1 ɥm. **b,** Spore titer of *gapA-ssrA* and *pyk-ssrA* mutants 24 hours after sporulation initiation (Supplementary Table 1). No sporulation phenotype was observed.

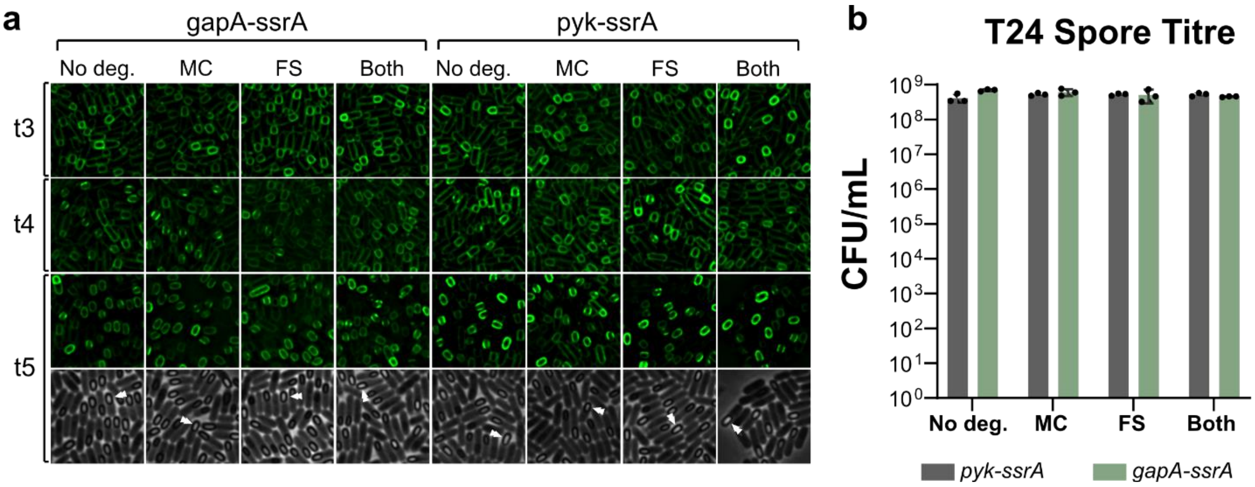

Supplementary Table 1. Spore titers

Tagged Protein	Degradation	Strain No.	Glutamate medium (spores/mL)
Cmk-ssrA	No Deg	KP1602	7.00E+08
	MC	BJAL81	5.13E+08
	FS	BJAL82	5.33E+08
	Both	BJAL86	3.73E+08
PyrG-ssrA (18 hours)	No Deg	KP1734	5.73E+08
	MC	BJAL83	6.07E+08
	FS	BJAL84	1.00E+05
	Both	BJAL85	9.33E+05
PyrG-ssrA (24 hours)	No Deg	KP1734	4.87E+08
	MC	BJAL83	4.80E+08
	FS	BJAL84	2.00E+07
	Both	BJAL85	3.07E+07
Cmk-ssrA, PyrG-ssrA	No Deg	BJAL70	4.40E+08
	MC	BJAL77	3.40E+04
	FS	BJAL78	0.00E+00
	Both	BJAL87	0.00E+00
Gmk-ssrA	No Deg	KP1638	3.00E+07
	MC	BJAL46	1.73E+04
	FS	BJAL47	0.00E+00
	Both	BJAL48	0.00E+00
GapA-ssrA	No Deg	BER0002	4.13E+08
	MC	BJAL142	5.26E+08
	FS	BER0021	5.26E+08
	Both	BJAL144	5.27E+08
Pyk-ssrA	No Deg	BER1722	7.00E+08
	MC	BER1784	6.13E+08
	FS	BER1785	5.07E+08
	Both	BER1786	4.53E+08

Supplementary Table 2. Strains used in this study.

Strain	Genotype or description	Reference, source or construction
PY79	Wild type	(Youngman et al., 1984)[1]
BER0002	*gapA-ssrAΩkan*	JLG974 → PY79
BER0021	*gapA-ssrAΩkan, amyE::PsspE(2G)-sspBΩcat*	BER0002 → BER0007
BER0083	*eno-gfp-ssrAΩkan*	pER013A → BER0001
BER0090	*gapA-gfp-ssrAΩkan*	pER012A → BER0001
BER0905	*citZ-gfp (unmarked)*	pCrePAΩerm → JLG2114
BER1052	*cmk-gfp-ssrAΩkan*	pER258a → PY79
BER1053	*gmk-gfp-ssrAΩkan*	pER259a → PY79
BER1098	*metE-gfpΩkan*	pER268a → PY79 (Riley et al 2021)[2]
BER131	*pelB::PcotE-sspBΩspec*	(Riley et al., 2018)[3]
BER1711	*ilvB-gfpΩkan*	pER398 → PY79
BER1715	*pckA-gfpΩkan*	pER403 → PY79
BER1718	*pdhD-gfpΩkan*	pER406 → PY79
BER1721	*pyk-gfpΩkan*	pER409 → PY79
BER1722	*pyk-ssrAΩkan*	pER410 → PY79
BER1784	*pyk-ssrAΩkan,, pelB::PcotE-sspBΩspec*	BER1722 → BER0007
BER1785	*pyk-ssrAΩkan, amyE::PsspE(2G)-sspBΩcat*	BER1722 → BER0131
BER1786	*pyk-ssrAΩkan, amyE::PsspE(2G)-sspBΩcat, pelB::PcotE-sspBΩspec*	BER1722 → BER0174
BER1863	*lutC-gfpΩkan*	pER426 → PY79

BER1874	*pyrK-gfpΩkan*	pER428 → PY79
BER1914	*argJ-gfpΩkan*	pER442 → PY79
BER1915	*argB-gfpΩkan*	pER443 → PY79
BER1916	*argD-gfpΩkan*	pER444 → PY79
BJAL047	*gmk-ssrAΩkan, amyE::PsspE(2G)-sspBΩcat*	KP1638 → BER0007
BJAL066	*cmk-ssrA (unmarked)*	Kan cassette excised from KP1602 with pCrePAΩerm plasmid
BJAL068	*pyrG-ssrA (unmarked)*	Kan cassette excised from KP1734 with pCrePAΩerm plasmid
BJAL070	*pyrG-ssrA (unmarked), cmk-ssrAΩkan*	KP1602 → BJAL068
BJAL072	*cmk-ssrA (unmarked), pyrG-ssrAΩkan*	KP1734 → BJAL066
BJAL078	*cmk-ssrA (unmarked), pyrG-ssrAΩkan, amyE::PsspE(2G)-sspBΩcat*	BER0007 → BJAL72
BJAL080	*pyrG-ssrA (unmarked), cmk-ssrAΩkan, amyE::PsspE(2G)-sspBΩcat*	BER0007 → BJAL070
BJAL082	*cmk-ssrAΩkan,amyE::PsspE(2G)-sspBΩcat*	KP1602 → BER0007
BJAL084	*pyrG-ssrAΩkan, amyE::PsspE(2G)-sspBΩcat*	KP1734 → BER0007
BJAL130	*gmk-ssrAΩkan, pelB::PcotE-sspBΩspec*	KP1638 → BER0131
BJAL132	*gmk-ssrAΩkan, pelB::PcotE-sspBΩspec, amyE::PsspE(2G)-sspBΩcat*	KP1638 → BER0174
BJAL134	*cmk-ssrAΩkan, pelB::PcotE-sspBΩspec*	KP1602 → BER0131
BJAL136	*cmk-ssrAΩkan, pelB::PcotE-sspBΩspec, amyE::PsspE(2G)-sspBΩcat*	KP1602 → BER0174
BJAL138	*pyrG-ssrAΩkan, pelB::PcotE-sspBΩspec*	KP1734 → BER0131
BJAL140	*pyrG-ssrAΩkan, pelB::PcotE-sspBΩspec, amyE::PsspE(2G)-sspBΩcat*	KP1734 → BER0174
BJAL142	*gapA-ssrAΩkan,, pelB::PcotE-sspBΩspec*	BER0002 → BER0131

BJAL144	*gapA-ssrAΩkan, amyE::PsspE(2G)-sspBΩcat, pelB::PcotE-sspBΩspec*	BER0002 → BER0174
BJAL146	*pyrG-ssrA (unmarked), cmk-ssrAΩkan, pelB::PcotE-sspBΩspec*	BER0131 → BJAL070
BJAL148	*cmk-ssrA (unmarked), pyrG-ssrAΩkan, pelB::PcotE-sspBΩspec*	BER0131 → BJAL072
BJAL150	*cmk-ssrA (unmarked), pyrG-ssrAΩkan, pelB::PcotE-sspBΩspec, amyE::PsspE(2G)-sspBΩcat*	BER0131 → BJAL078
BJAL152	*pyrG-ssrA (unmarked), cmk-ssrAΩkan, pelB::PcotE-sspBΩspec, amyE::PsspE(2G)-sspBΩcat*	BER0131 → BJAL080
BKR739	*scoB-gfpΩkan*	pKR76 → PY79
JLG963	*amyE::PsspE(2G)-sspB*^{Ec}*Ωcat*	(Lopez-Garrido et al., 2018)[4]
KP1602	*cmk-ssrAΩkan*	pJLG109 → PY79
KP1638	*gmk-ssrAΩkan*	pJLG136 → PY79
KP1734	*pyrG-ssrAΩkan*	pJLG176 → PY79

Supplementary Table 3. Plasmids used in this study.

Plasmid	Description	Reference
pCrePA	pCreΩerm	(Pomerantsev et al., 2006)[5]
pDG1662	*plasmid backbone*	(Guérout-Fleury et al., 1996)[6]
pER012A	*gapA-gfp-ssrAΩkan*	This study
pER013A	*eno-gfp-ssrAΩkan*	This study
pER258a	*cmk-gfp-ssrAΩkan*	This study
pER259a	*gmk-gfp-ssrAΩkan*	This study
pER268a	*metE-gfpΩkan*	This study
pER398	*ilvB-gfpΩkan*	This study
pER403	*pckA-gfpΩkan*	This study
pER406	*pdhD-gfpΩkan*	This study
pER409	*pyk-gfpΩkan*	This study
pER410	*pyk-ssrAΩkan*	This study
pER426	*lutC-gfpΩkan*	This study
pER428	*pyrK-gfpΩkan*	This study
pER442	*argJ-gfpΩkan*	This study
pER443	*argB-gfpΩkan*	This study
pER444	*argD-gfpΩkan*	This study
pKR76	*scoB-gfpΩkan*	This study

Supplementary Table 4. Primers used in this study.

Primer	Sequence
oER0042	gcgcttgcgctgctagcAAGACCTTTTTTTGCGATGTAAG
oER0043	gcgcttgcgctgctagcCTTGTTTAAGTTGTAGAAAGAGTTGATACC
oER0044	ggcatggatgaactgtataaaGCTAGCGCAGCAAATGATG
oER1389	gggttaacgcgtaatccatgGTAGGCCAGCATCAAATGTGG
oER1390	gcgcttgcgctgctagcAGGTTTCACCCCCACCATTTC
oER1391	cactggagttgtcccaattTTGAAAAGAATTATCACATTGACTGTG
oER1392	cacatttccccgaaaagtgcCTTTTACCATTTCAATCTCTCCC
oER1408	gggttaacgcgtaatccatgCCTCGTATCTGGCGTATTCGC
oER1410	cactggagttgtcccaattTTACAGGTGAAAATGGAAGGG
oER1411	cacatttccccgaaaagtgcCCGCTTATTCTTCGACATTCC

oER1412	gcgcttgcgctgctagcAAGAACGCTCGCACGGC
oER1415	gggttaacgcgtaatccatgGGTATCGAAATGACGGACC
oER1417	cactggagttgtcccaattTTTTCATATCAAAAACAGCCCC
oER1418	cacatttccccgaaaagtgcCTTTCATCCAAGAATGCAGC
oER1419	gcgcttgcgctgctagcTTTTACGATGTGAATCGGAC
oER1466	gggttaacgcgtaatccatgCTGATTGAAACGGATTCAACCG
oER1467	gcgcttgcgctgctagcGCGGTCAGAGACGAGAATATATG
oER1468	cactggagttgtcccaattACTCAGGAAGCCCGGCAG
oER1469	cacatttccccgaaaagtgcTGAATGAAGTTCGAGTATGCAGCG
oER1484	gggttaacgcgtaatccatgGAGAACTTGTGGATGTCCTCG
oER1485	gcgcttgcgctgctagcCAGCGCCACCTCCTGAG
oER1486	cactggagttgtcccaattATGCTAGAGGTGAAATTGCCG
oER1487	cacatttccccgaaaagtgcTGTGATATTAGCCACGTTCGG
oER1533	gggttaacgcgtaatccatgACATCCACGAACGACATGG
oER1534	gcgcttgcgctgctagcCGTGCGATAGCTCGCG
oER1535	cactggagttgtcccaattTAAAGGGGAGCGAAATGAAG
oER1536	cacatttccccgaaaagtgcTTCATGATTCCATCGACATCC
oER1539	gggttaacgcgtaatccatgTTTCGTTGCTGAGCTTGC
oER1540	gcgcttgcgctgctagcTGAAACAGCCTCCTTTGC
oER1541	cactggagttgtcccaattAAAGGAGGCTGTTTCATGAG
oER1542	cacatttccccgaaaagtgcAAGCAGAAGGATCATTGTATGG
oER1557	gggttaacgcgtaatccatgGTCGTTTTGCAAGGAAAAGC
oER1558	gcgcttgcgctgctagcCTGGTTTACAGCGGAATGATG
oER1559	cactggagttgtcccaattTTTTTTTTCGATATAAAGGCATAAAAATTC
oER1560	cacatttccccgaaaagtgcTGAGAGCGATATGTTTATTCCC
oJLG007	aattgggacaactccagtg
oJLG077	GCTAGCagcgcaagcgc
oJLG086	TAAATGAGAGAGGAAGAAAAcGG
oJLG087	TCATttAtacagTtcAtccatgcc
oJLG095	catggattacgcgttaaccc
oJLG096	gcacttttcggggaaatgtg
oJLG498	TCAGCATCATCAgcaagcgcaGATGAGAGAGGAAGAAAAcGG
oKR217	gggttaacgcgtaatccatgCATGCTTCAGTCGGAAAACG
oKR219	cactggagttgtcccaattcTGGGAAGGTGTTTGACATGAG
oKR220	cacatttccccgaaaagtgcGCTGGGTATCGACATAACCC
oKR221	cttgcgcttgcgctgctagcAGAATTGAGTACAGACTGGCT

Supplementary Methods

Plasmid construction

pER012A
This plasmid was constructed by assembling 2 fragments using Gibson Assembly (New England Biolads): (i) inverse PCR DNA fragment of plasmid pJLG139 using primers oER42 and oER44, and (ii) gfp fragment amplified from pJLG38 with primers JLG-77 and JLG-498.

pER013A
This plasmid was constructed by assembling the following fragments using Gibson Assembly (New England Biolads): (i) inverse PCR DNA fragment of plasmid from pER001a using primers oER43 and oER44, and (ii) gfp fragment amplified from pJLG38 with primers JLG-77 and JLG-498.

pER258a
This plasmid was constructed by the ligation of 2 restriction enzyme digested fragments: (i) a 6326 bp fragment digested from plasmid pJLG109 with NheI and BAMHI, and (ii) a 807 bp fragment digested from plasmid pER252A with NheI and BAMHI.

pER259a
This plasmid was constructed by the ligation of 2 restriction enzyme digested fragments: (i) a 6232 bp fragment digested from plasmid pJLG136 with NheI and BAMHI, and (ii) a 807 bp fragment digested from plasmid pER252A with NheI and BAMHI.

pER268a
This plasmid was constructed by ligating the following fragment: Reverse PCR of plasmid pER175a using phosphorylated oligos JLG-86 and JLG-87. The PCR product was DpnI digested and ligated.

pER398
This plasmid was constructed by the assembly of the following 4 fragments using Gibson Assembly (New England Biolabs): (i) 3' region of *ilvB* coding sequence (not including the stop codon) amplified with primers oER1389 and oER1390 from genomic DNA of *B. subtilis* PY79, (ii) *gfp**Ωkan* amplified from plasmid pJLG38 (Yen Shin et al., 2015) using primers JLG-7 and JLG-77, (iii) region immediately downstream *ilvB* stop codon, amplified with primers oER1391 and oER1392, (iv) a DNA fragment encompassing the spectinomycin resistant gene, the origin of replication, and the ampicillin resistant gene from pDG1662 (Guérout-Fleury et al., 1996), amplified with primers JLG-95 and JLG-96.

pER403
This plasmid was constructed by the assembly of the following 4 fragments using Gibson Assembly (New England Biolabs): (i) 3' region of *pckA* coding sequence (not including the stop codon) amplified with primers oER1401 and oER1405 from genomic DNA of *B. subtilis* PY79, (ii) *gfp**Ωkan* amplified from plasmid pJLG38 (Yen Shin et al., 2015)[7] using primers JLG-7 and JLG-77, (iii) region immediately downstream *pckA* stop codon, amplified with primers oER11403 and oER1404, (iv) a DNA fragment encompassing the spectinomycin resistant gene,

the origin of replication, and the ampicillin resistant gene from pDG1662 (Guérout-Fleury et al., 1996)[6], amplified with primers JLG-95 and JLG-96.

pER406
This plasmid was constructed by the assembly of the following 4 fragments using Gibson Assembly (New England Biolabs): (i) 3’ region of *pdhD* coding sequence (not including the stop codon) amplified with primers oER1415 and oER1419 from genomic DNA of *B. subtilis* PY79, (ii) *gfp*Ωkan* amplified from plasmid pJLG38 (Yen Shin et al., 2015)[7] using primers JLG-7 and JLG-77, (iii) region immediately downstream *pdhD* stop codon, amplified with primers oER1403 and oER1404, (iv) a DNA fragment encompassing the spectinomycin resistant gene, the origin of replication, and the ampicillin resistant gene from pDG1662 (Guérout-Fleury et al., 1996)[6], amplified with primers JLG-95 and JLG-96.

pER409
This plasmid was constructed by the assembly of the following 4 fragments using Gibson Assembly (New England Biolabs): (i) 3’ region of *pyk* coding sequence (not including the stop codon) amplified with primers oER1408 and oER1412 from genomic DNA of *B. subtilis* PY79, (ii) *gfp*Ωkan* amplified from plasmid pJLG38 (Yen Shin et al., 2015)[7] using primers JLG-7 and JLG-77, (iii) region immediately downstream *pyk* stop codon, amplified with primers oER1410 and oER1411, (iv) a DNA fragment encompassing the spectinomycin resistant gene, the origin of replication, and the ampicillin resistant gene from pDG1662 (Guérout-Fleury et al., 1996)[6], amplified with primers JLG-95 and JLG-96.

pER410
This plasmid was constructed by the assembly of the following 4 fragments using Gibson Assembly (New England Biolabs): (i) 3’ region of *pyk* coding sequence (not including the stop codon) amplified with primers oER1408 and oER1412 from genomic DNA of *B. subtilis* PY79, (ii) *ssrA*Ωkan* amplified from plasmid pJLG38 (Yen Shin et al., 2015)[7] using primers JLG-7 and JLG-77, (iii) region immediately downstream *pyk* stop codon, amplified with primers oER1410 and oER1411, (iv) a DNA fragment encompassing the spectinomycin resistant gene, the origin of replication, and the ampicillin resistant gene from pDG1662 (Guérout-Fleury et al., 1996)[6], amplified with primers JLG-95 and JLG-96.

pER426
This plasmid was constructed by the assembly of the following 4 fragments using Gibson Assembly (New England Biolabs): (i) 3’ region of *lutC* coding sequence (not including the stop codon) amplified with primers oER1466 and oER1467 from genomic DNA of *B. subtilis* PY79, (ii) *gfp*Ωkan* amplified from plasmid pJLG38 (Yen Shin et al., 2015)[7] using primers JLG-7 and JLG-77, (iii) region immediately downstream *lutC* stop codon, amplified with primers oER1468 and oER1469, (iv) a DNA fragment encompassing the spectinomycin resistant gene, the origin of replication, and the ampicillin resistant gene from pDG1662 (Guérout-Fleury et al., 1996)[6], amplified with primers JLG-95 and JLG-96.

pER428
This plasmid was constructed by the assembly of the following 4 fragments using Gibson Assembly (New England Biolabs): (i) 3’ region of *pyrK* coding sequence (not including the stop

codon) amplified with primers oER1484 and oER1485 from genomic DNA of *B. subtilis* PY79, (ii) *gfp*Ωkan* amplified from plasmid pJLG38 (Yen Shin et al., 2015)[7] using primers JLG-7 and JLG-77, (iii) region immediately downstream *pyrK* stop codon, amplified with primers oER14486 and oER1487, (iv) a DNA fragment encompassing the spectinomycin resistant gene, the origin of replication, and the ampicillin resistant gene from pDG1662 (Guérout-Fleury et al., 1996)[6], amplified with primers JLG-95 and JLG-96.

pER442
This plasmid was constructed by the assembly of the following 4 fragments using Gibson Assembly (New England Biolabs): (i) 3' region of *argJ* coding sequence (not including the stop codon) amplified with primers oER1533 and oER1534 from genomic DNA of *B. subtilis* PY79, (ii) *gfp*Ωkan* amplified from plasmid pJLG38 (Yen Shin et al., 2015) using primers JLG-7 and JLG-77, (iii) region immediately downstream *argJ* stop codon, amplified with primers oER1535 and oER1536, (iv) a DNA fragment encompassing the spectinomycin resistant gene, the origin of replication, and the ampicillin resistant gene from pDG1662 (Guérout-Fleury et al., 1996)[6], amplified with primers JLG-95 and JLG-96.

pER443
This plasmid was constructed by the assembly of the following 4 fragments using Gibson Assembly (New England Biolabs): (i) 3' region of *argB* coding sequence (not including the stop codon) amplified with primers oER1539 and oER1540 from genomic DNA of *B. subtilis* PY79, (ii) *gfp*Ωkan* amplified from plasmid pJLG38 (Yen Shin et al., 2015) using primers JLG-7 and JLG-77, (iii) region immediately downstream *argB* stop codon, amplified with primers oER1541 and oER1542, (iv) a DNA fragment encompassing the spectinomycin resistant gene, the origin of replication, and the ampicillin resistant gene from pDG1662 (Guérout-Fleury et al., 1996)[6], amplified with primers JLG-95 and JLG-96.

pER444
This plasmid was constructed by the assembly of the following 4 fragments using Gibson Assembly (New England Biolabs): (i) 3' region of *argD* coding sequence (not including the stop codon) amplified with primers oER1557 and oER1558 from genomic DNA of *B. subtilis* PY79, (ii) *gfp*Ωkan* amplified from plasmid pJLG38 (Yen Shin et al., 2015) using primers JLG-7 and JLG-77, (iii) region immediately downstream *argD* stop codon, amplified with primers oER1559 and oER1560, (iv) a DNA fragment encompassing the spectinomycin resistant gene, the origin of replication, and the ampicillin resistant gene from pDG1662 (Guérout-Fleury et al., 1996)[6], amplified with primers JLG-95 and JLG-96.

pKR76
This plasmid was constructed by the assembly of the following 4 fragments using Gibson Assembly (New England Biolabs): (i) 3' region of *scoB* coding sequence (not including the stop codon) amplified with primers oKR217 and oKR221 from genomic DNA of *B. subtilis* PY79, (ii) *gfp-ssrA*Ωkan* amplified from plasmid pJLG12 using primers JLG-7 and JLG-77, (iii) region immediately downstream *scoB* stop codon, amplified with primers oKR219 and oKR220, (iv) a DNA fragment encompassing the spectinomycin resistant gene, the origin of replication, and the ampicillin resistant gene from pDG1662 (Guérout-Fleury et al., 1996)[6], amplified with primers JLG-95 and JLG-96.

pER001a

This plasmid was constructed by the assembly of the following 4 fragments using Gibson Assembly (New England Biolabs): (i) 3’ region of *eno* coding sequence (not including the stop codon) amplified with primers JLG-561 and JLG-562 from genomic DNA of *B. subtilis* PY79, (ii) *ssrA**Ωkan* amplified from plasmid pJLG3 using primers JLG-184 and JLG-7, (iii) region immediately downstream *eno* stop codon, amplified with primers JLG-563 and JLG-564, (iv) a DNA fragment encompassing the spectinomycin resistant gene, the origin of replication, and the ampicillin resistant gene from pDG1662 (Guérout-Fleury et al., 1996)[6], amplified with primers JLG-95 and JLG-96.

pER175a

This plasmid was constructed by the assembly of the following 4 fragments using Gibson Assembly (New England Biolabs): (i) 3’ region of *metE* coding sequence (not including the stop codon) amplified with primers oER614 and oER618 from genomic DNA of *B. subtilis* PY79, (ii) *gfp-ssrA**Ωkan* amplified from plasmid pJLG3 using primers JLG-77 and JLG-7, (iii) region immediately downstream *metE* stop codon, amplified with primers oER616 and oER617, (iv) a DNA fragment encompassing the spectinomycin resistant gene, the origin of replication, and the ampicillin resistant gene from pDG1662 (Guérout-Fleury et al., 1996)[6], amplified with primers JLG-95 and JLG-96.

pER252A

This plasmid was constructed by the assembly of the following 4 fragments using Gibson Assembly (New England Biolabs): (i) 3’ region of *thyA* coding sequence (not including the stop codon) amplified with primers oER1009 and oER1013 from genomic DNA of *B. subtilis* PY79, (ii) *gfp-ssrA**Ωkan* amplified from plasmid pJLG3 using primers JLG-77 and JLG-7, (iii) region immediately downstream *thyA* stop codon, amplified with primers oER1011 and oER1012, (iv) a DNA fragment encompassing the spectinomycin resistant gene, the origin of replication, and the ampicillin resistant gene from pDG1662 (Guérout-Fleury et al., 1996)[6], amplified with primers JLG-95 and JLG-96.

pJLG12

loxPKmloxP amplified from pEB19 wih primers JLG-5 and JLG-42, digested with NheI and PstI, and cloned in pBR329 digested with NheI and PstI. It contains two stops codons in frame with NheI restriction site, to stop translation of genes cloned upstream of it.

pJLG109

This plasmid was constructed by the assembly of the following 4 fragments using Gibson Assembly (New England Biolabs): (i) 3’ region of *cmk* coding sequence (not including the stop codon) amplified with primers JLG-398 and JLG-399 from genomic DNA of *B. subtilis* PY79, (ii) *AALGG**Ωkan* amplified from plasmid pJLG3 using primers JLG-184 and JLG-7, (iii) region immediately downstream *cmk* stop codon, amplified with primers JLG-400 and JLG-401, (iv) a DNA fragment encompassing the spectinomycin resistant gene, the origin of replication, and the ampicillin resistant gene from pDG1662 (Guérout-Fleury et al., 1996)[6], amplified with primers JLG-95 and JLG-96.

pJLG136
This plasmid was constructed by the assembly of the following 4 fragments using Gibson Assembly (New England Biolabs): (i) 3' region of *gmk* coding sequence (not including the stop codon) amplified with primers JLG-591 and JLG-592 from genomic DNA of *B. subtilis* PY79, (ii) *AALGG*Ωkan* amplified from plasmid pJLG3 using primers JLG-184 and JLG-7, (iii) region immediately downstream *gmk* stop codon, amplified with primers JLG-593 and JLG-594, (iv) a DNA fragment encompassing the spectinomycin resistant gene, the origin of replication, and the ampicillin resistant gene from pDG1662 (Guérout-Fleury et al., 1996)[6], amplified with primers JLG-95 and JLG-96.

pJLG139
This plasmid was constructed by the assembly of the following 4 fragments using Gibson Assembly (New England Biolabs): (i) 3' region of *gapA* coding sequence (not including the stop codon) amplified with primers JLG-573 and JLG-574 from genomic DNA of *B. subtilis* PY79, (ii) *AALGG*Ωkan* amplified from plasmid pJLG3 using primers JLG-184 and JLG-7, (iii) region immediately downstream *gapA* stop codon, amplified with primers JLG-575 and JLG-576, (iv) a DNA fragment encompassing the spectinomycin resistant gene, the origin of replication, and the ampicillin resistant gene from pDG1662 (Guérout-Fleury et al., 1996)[6], amplified with primers JLG-95 and JLG-96.

References

1. Youngman, P., Perkins, J. B. & Losick, R. Construction of a cloning site near one end of Tn917 into which foreign DNA may be inserted without affecting transposition in *Bacillus subtilis* or expression of the transposon-borne *erm* gene. *Plasmid* **12**, 1–9 (1984).
2. Riley, E. P., Lopez-Garrido, J., Sugie, J., Liu, R. B. & Pogliano, K. Metabolic differentiation and intercellular nurturing underpin bacterial endospore formation. *Sci. Adv.* **7**, eabd6385 (2021).
3. Riley, E. P. *et al.* Spatiotemporally regulated proteolysis to dissect the role of vegetative proteins during *Bacillus subtilis* sporulation: cell-specific requirement of σH and σA. *Mol. Microbiol.* **108**, 45–62 (2018).
4. Lopez-Garrido, J. *et al.* Chromosome translocation inflates bacillus forespores and impacts cellular morphology. *Cell* **172**, 758-770.e14 (2018).
5. Pomerantsev, A. P., Sitaraman, R., Galloway, C. R., Kivovich, V. & Leppla, S. H. Genome engineering in *Bacillus anthracis* using Cre recombinase. *Infection and Immunity* **74**, 682–693 (2006).
6. Guérout-Fleury, A. M., Frandsen, N. & Stragier, P. Plasmids for ectopic integration in Bacillus subtilis. *Gene* **180**, 57–61 (1996).
7. Yen Shin, J. *et al.* Visualization and functional dissection of coaxial paired SpoIIIE channels across the sporulation septum. *Elife* **4**, e06474 (2015).
