# Supplementary material for: Deciphering metabolic differentiation during Bacillus subtilis sporulation
Source: Nat Commun. 2025 Jan 2;16:129. doi: 10.1038/s41467-024-55586-z (PMC11695771; doi:10.1038/s41467-024-55586-z)
Supplement: Supplementary file 3 — Description of Additional Supplementary Files [file 41467_2024_55586_MOESM3_ESM.pdf]

Description of Additional Supplementary file

Supplementary Data 1. Complete flux distributions predicted by SporeME2.

Supplementary Data 2. Cellular location of the main reactions predicted to provide amino acids to the forespore.

Supplementary Data 3. Protein essentiality predictions in the mother cell, forespore, and vegetative cell, and their intersections. Predictions are labeled TRUE if the protein was predicted to be required in each cell and FALSE if it was not predicted to be required. Each Supplementary Data sheet shows a different intersection, following the partitions shown in Fig.4A.

Supplementary Data 4. Pathway enrichment analysis of proteins predicted to be required in the mother cell, forespore and vegetative cell. This dataset includes the pathway enrichment results by DAVID of predicted required protein lists in every cell and their intersections.

Supplementary Data 5. Protein essentiality interdependence predictions by SporeME2. Predictions are represented as a directed graph, where an edge denotes that the source node either inactivates or blocks the target node. Every node is marked according to whether it has been suggested by mass spectrometry or confirmed by GFP tagging.

Supplementary Data 6. Protein essentiality interdependence predictions by the naïve SporeME2. Constrained depletions in the forespore were reverted in SporeME2 to assess their effect on predictions. Predictions are represented as a directed graph, where an edge denotes that the source node either inactivates or blocks the target node. Each node is marked according to whether it has been suggested by mass spectrometry or confirmed by GFP tagging.
